# Parenting behavior in families of female adolescents with nonsuicidal self-injury in comparison to a clinical and a nonclinical control group

**DOI:** 10.1186/s13034-015-0051-x

**Published:** 2015-07-08

**Authors:** Taru Tschan, Marc Schmid, Tina In-Albon

**Affiliations:** University of Koblenz-Landau, Clinical Child and Adolescent Psychology and Psychotherapy, Ostbahnstrasse 12 76829, Landau, Germany; Department of Child and Adolescent Psychiatry, University of Basel, Basel, Switzerland

**Keywords:** Nonsuicidal self-injury (NSSI), Parenting behavior, Parent–child interaction, Warmth and support

## Abstract

**Background:**

Nonsuicidal self-injury (NSSI) is often accompanied by dysfunctional familial relationships. Problems within the family are also frequent triggers for NSSI.

**Methods:**

The current study investigated the parenting behavior in families of 45 female adolescents with NSSI disorder, 27 adolescents with other mental disorders (clinical controls, CCs), and 44 adolescents without mental disorders (nonclinical controls, NCs). The adolescents and their parents (92 mothers, 24 fathers) were surveyed using self-report measures. The parenting dimensions warmth and support, psychological control, and behavioral control (demands, rules, and discipline), as well as parental psychopathology and parental satisfaction were assessed.

**Results:**

Adolescents with NSSI disorder reported significantly less maternal warmth and support than NCs (*d* = .64); this group difference was not evident in mothers’ reports. No group differences emerged regarding adolescent-reported paternal parenting behavior. Mothers of adolescents with NSSI reported higher depression, anxiety, and stress scores than mothers in the NC group and less parental satisfaction than mothers in both control groups (CC and NC).

**Conclusions:**

Given the association between NSSI, low levels of adolescent-reported maternal warmth and support and low levels of mother-reported parental satisfaction, clinical interventions for adolescents with NSSI should focus on improving family communication and interaction.

## Introduction

Nonsuicidal self-injury (NSSI) has been included in the fifth edition of the *Diagnostic and Statistical Manual of Mental Disorders (DSM-5)* [1] as a condition requiring further study. NSSI disorder is defined as the direct and intentional injury of one’s own body tissue without suicidal intent [1, 2]. The 6-month prevalence rate for single NSSI ranges between 7.6 and 14.6 % in Austria, Germany, and Switzerland [3]. The prevalence rate for repetitive NSSI using the criteria of the *DSM-5* [1] was 6.7 % in a recent community study [4].

Research has shown that NSSI principally serves an intrapersonal function. Adolescents engage in NSSI to cope with negative thoughts and feelings [5–7]. Nevertheless, intense negative emotions are often caused by negative interpersonal interactions and experiences. Therefore, interpersonal processes also play an important role, especially in the onset and maintenance of NSSI [8]. According to Vonderlin et al. [9], adolescents with NSSI often report relationship problems with relatives and peers. Problems concerning family and peer relationships, self-worth, alcohol and drug consumption, and experiences of loss and violence were more common among adolescents with NSSI than adolescents without NSSI in a school sample [9]. Whether these interpersonal difficulties are possible antecedents or consequences of NSSI has not yet been determined [10].

Linehan [11] posited that an invalidating family environment might influence the onset of NSSI. The characteristics of an invalidating family environment are inadequate parenting and family functioning. The relationships with caregivers are distinguished by a high level of negativity and control and a lack of support. The communication of personal experiences is not validated; instead it is often disregarded, trivialized, or punished. An invalidating environment can lead to deficits in emotion regulation and thus increase the likelihood of adopting negative skills (e.g., NSSI). Consistent with Linehan’s theory, research has shown associations between an invalidating family environment and the development and maintenance of NSSI e.g., [12, 13].

Adverse childhood experiences, especially maternal antipathy and neglect, are highly associated with NSSI [13]. Previous findings indicate higher levels of negative affect and lower levels of positive affect and cohesiveness in families of adolescents with self-injurious behavior [12]. The absence of a family confidant and poorer family communication were found to be associated with adolescent self-injury [14]. High parental expressed emotion, especially criticism, was associated with adolescents’ NSSI. The relationship between parental expressed emotion and NSSI was strong in particular among adolescents with a self-critical cognitive style [15]. Fruzzetti, Santisteban, and Hoffman [16] described a complex interaction between a patient with severe problems in emotion regulation and the reaction of family members to the child’s behavior. This interaction is understood as a combination of the high expressed emotion concept [17, 18] and Linehan’s [11] psychosocial theory of emotion regulation. Obviously, family members need a high capacity to regulate their own emotions to communicate effectively with the affected family member. The relationship between parental psychopathology, parental stress, and insufficient or maladaptive parent–child interaction has been well established [19, 20]. It is important to consider the vicious circle of insufficient parent–child interactions, the symptoms of the child and the parent, and the parental sense of being considerably burdened by caring for an adolescent with NSSI. Compared to adolescents without NSSI, adolescents engaging in NSSI have described their relationships with their parents as being characterized by less trust, less communication, and more alienation [21]. This is in line with Bureau et al.’s [22] finding that the parent–child relationships of adolescents with NSSI are characterized by failed protection, much control, and feelings of alienation. Adolescents with NSSI perceive more psychological and behavioral control from their parents than adolescents without NSSI [23].

Baetens et al. [23] did not find any differences in parent-reported parenting stress. Morgan et al. [24] reported that the majority of parents of adolescents with NSSI showed low levels of well-being, parental satisfaction, and social support. Mother’s mental distress and health problems were found to predict self-harm in adolescents [19].

Existing studies indicate that family experiences can influence the onset and maintenance of NSSI. However, to our knowledge, no study has investigated parenting behavior in adolescents with NSSI that fits *DSM-5* criteria [22, 23]. Instead, NSSI has been assessed using single-item measures [21, 23] and different questionnaires [12, 15, 22]. Different types of assessment contribute to there being different estimates of the prevalence of NSSI [8] and may also assess different adolescents. To determine the frequency and severity of self-injurious behavior, other studies have taken into account either the whole life span [10] or the past 6–12 months [15, 22]. Therefore, the studies are not comparable regarding the actual frequency of NSSI. Previous studies investigated both clinical [10] and nonclinical samples [22, 23] and thus differ regarding the adolescents’ psychopathology and the severity of the examined NSSI. Students with a single episode of NSSI are possibly not representative of the whole group of adolescents with NSSI [25]. In the nonclinical studies [22, 23], no structured clinical interviews were conducted for the group assignments of adolescents with and without NSSI. Therefore, inaccurate group assignment and disregard for comorbid disorders cannot be excluded. Differentiating between diagnoses of NSSI and borderline personality disorder (BPD) is especially important, as only about one third of adolescents with NSSI also meet criteria for BPD [26].

So far, it can be stated that adolescents with NSSI perceive more unfavorable parenting behavior than adolescents without NSSI [21, 22]. Only one study [23] examined both adolescent- and parent-reports on parenting behaviors. Therefore, in the present study we investigated the parenting behavior in families of adolescents with NSSI, adolescents with other mental disorders (clinical controls), and adolescents without mental disorders (nonclinical controls). The three groups were compared regarding the parenting behaviors warmth and support, psychological control, and behavioral control. We used self-report measures to assess the parenting behavior from the parents’ and adolescents’ perspective. Taking the results of previous studies into account, we hypothesized that adolescents with NSSI disorder would report less warmth and support, more psychological control, and less behavioral control (demands, rules, discipline) in the relationship with their parents than both the CC and the NC group. Furthermore, we examined parent–adolescent agreement regarding parenting behaviors as well as parental psychopathology and parental stress. We hypothesized that parents of adolescents with NSSI disorder would report more psychopathology and stress.

## Method

### Participants

Participants were 116 female adolescents (ages 13–20 years, *M* = 16.01; *SD* = 1.64). The sample included 45 adolescents with NSSI disorder, 27 adolescents with other mental disorders without NSSI (clinical controls, CCs), and 44 adolescents without current or past experience of mental disorders (nonclinical controls, NCs). Participants were similar with respect to age, *F*(2, 112) = 2.93, *p* > .05.

All adolescents were diagnosed using the *Diagnostic Interview for Mental Disorders in Children and Adolescents* (Kinder-DIPS) [27], a structured interview in German based on the *DSM-IV-TR* criteria [28].

#### Diagnostic characteristics

The mean number of diagnoses was 3.36 (*SD* = 1.42) for adolescents with NSSI and 2.07 (*SD* = 0.92) for CC adolescents, which is a significant difference, *t*(70) = 7.27, *p* < .01. The most frequent diagnosis among adolescents with NSSI and CC adolescents was major depression, followed by social phobia. Posttraumatic stress disorder was diagnosed more often in the NSSI group (*n* = 10, 22.2 %) than in the CC group (*n* = 2, 7.4 %), and borderline personality disorder (*n* = 7, 15.6 %) and alcohol abuse (*n* = 2, 4.4 %) emerged only in the NSSI group.

#### Family characteristics

A total of 116 parents including 92 mothers (ages 36–57 years, *M* = 45.67; *SD* = 4.91) and 24 fathers (ages 44–58 years, *M* = 48.74; *SD* = 3.13) participated. Participating fathers were significantly older than participating mothers, *F*(1, 103) = 7.79, *p* < .01. Parents’ education was assessed with the following scale: 0 (did not finish school), 1 (obligatory school), 2 (vocational training), 3 (*Matur*; slightly higher than a high school diploma), 4 (professional training), and 5 (university degree). Mothers’ mean education was 2.52 (*SD* = 1.23) in the NSSI group, 2.26 (*SD* = .87) in the CC group, and 3.12 (*SD* = 1.27) in the NC group, with a significant difference between the groups, *F*(2, 82) = 3.83, *p* < .05. Post hoc analyses indicated that this difference emerged between the CC and NC group. Fathers’ mean education was 4.00 (*SD* = .87) in the NSSI group, 4.75 (*SD* = .50) in the CC group, and 3.40 (*SD* = 1.51) in the NC group, with no significant difference between the groups, *F*(2,22) = 2.01, *p* > .05. The families’ average monthly income was assessed using a scale ranging from 1 (less than 2,000 Swiss francs per month) to 6 (more than 10,000 Swiss francs per month), with 2 = 2,000–4,000 and 3 = 4,001–6,000 Swiss francs per month. The mean income was 2.70 (*SD* = 1.45) in the NSSI group, 2.27 (*SD* = 1.03) in the CC group, and 2.23 (*SD* = 1.22) in the NC group, with no significant difference between the groups, *F*(2,82) = 1.26, *p* = .29.

### Procedure

The recruitment took place in Switzerland and Germany. The two clinical groups were recruited from different inpatient child and adolescent psychiatric units and the NC group from different schools. The inpatient clinics were responsible for the recruitment of the clinical groups. Therefore, we have no access to the demographic and clinical characteristics of patients excluded by the clinics. Our predefined exclusion criteria were current or past psychosis, schizophrenic symptoms, and acute substance abuse. The inpatient clinics were instructed to inform the participants at admission; in most cases it was not the therapist who did so. Adolescents and parents gave their written consent. The institutional review board (Ethikkommission beider Basel, EKBB) approved the study. Questionnaires were administered to the participating adolescents (Zurich Short Questionnaire on Parental Behavior, ZKE) and their parents (Depression Anxiety Stress Scale-21, DASS-21; Parental Stress Scale, PSS; Zurich Short Questionnaire on Parental Behavior, ZKE). The adolescents were paid 40 Swiss francs for participation.

### Measures

#### Assessment of Axis I and Axis II diagnoses

To examine current and past *DSM-IV-TR* diagnoses a structured interview for mental disorders in children and adolescents [27] was conducted with each adolescent. The Kinder-DIPS assesses the most frequent mental disorders in childhood and adolescence, including anxiety disorders, depression, attention-deficit/hyperactivity disorder, conduct disorder, sleep disorders, and eating disorders. The interview has good validity and reliability [29, 30]. NSSI disorder was assessed with an interview using the *DSM-5* criteria. The estimates of interrater reliability for the diagnosis of NSSI are very good (κ = 0.90) [26]. Questions about triggers for NSSI were part of the sociodemographic questionnaire. Substance use disorder and borderline personality disorder were examined with the adult DIPS [31]. Axis II personality disorders were obtained with the Structured Clinical Interview for DSM-IV Axis II Personality Disorders (SKID-II) [32].

### Depression Anxiety Stress Scale-21 (DASS-21)

This 21-item questionnaire assesses depression, anxiety, and stress symptoms [33]. Participants rate the frequency and severity of the symptoms over the last week on a 4-point Likert scale. The DASS-21 has a good internal consistency and convergent and discriminant validity [34]. The internal consistency in the present sample was α = 0.92 for the depression scale, α = 0.86 for the anxiety scale, α = 0.86 for the stress scale, and α = 0.95 for the total scale.

### Parental Stress Scale (PSS)

This instrument assesses parent satisfaction [35]. It contains items representing positive themes of parenthood such as emotional benefits or self-enrichment and negative components such as demands on resources and restrictions. The questionnaire consists of the four subscales parental rewards, parental stressors, lack of control, and parental satisfaction. The PSS has satisfactory levels of internal consistency and convergent and discriminant validity [35]. The internal consistency in the present sample was α = 0.76 for parental rewards, α = 0.51 for parental stressors, α = 0.68 for lack of control, and α = 0.59 for parental satisfaction.

### Parenting Behavior

The Zurich Short Questionnaire on Parental Behavior (ZKE) [36] assesses three aspects of parenting behavior from the parents’ and children’s perspective. Adolescents complete the questionnaire once for their mother and once for their father. The ZKE measures warmth and support, psychological pressure, and behavioral control (demands, rules, and discipline). The questionnaire demonstrated good psychometric properties. The internal consistency in the present sample was α = 0.93 for the subscale warmth and support, α = 0.88 for the subscale psychological pressure, and α = 0.72 for the subscale behavioral control.

### Data analysis

Data were checked to insure that they met the assumptions for the analyses; no violations of assumptions were detected. We used multivariate analysis of variance (MANOVA) to investigate group differences in parenting behavior, parental psychopathology, and parental stress between the groups. Post hoc tests were conducted to analyze pairwise comparisons (NSSI vs. CC, NSSI vs. NC, and CC vs. NC). The Bonferroni–Holm correction was used to control for multiple comparisons. Effect sizes (Cohen’s *d*) are used to report differences between the groups. An effect size of 0.20 equates to a small effect, 0.50 to a medium effect, and 0.80 to a large effect. Parent–child agreement regarding parenting behavior was evaluated by calculating Pearson product–moment correlation coefficients. To compare correlations the coefficients were converted to *z* scores. Analyses were performed using SPSS version 21. Significance levels were set at α = 0.05.

## Results

### Parenting behavior

Frequent triggers for NSSI were conflicts within the family (80 %) and with friends (48.9 %). The means and standard deviations of the ZKE on parenting behavior are reported in Table 1. Results of the MANOVA revealed a marginally significant difference between the groups in adolescent-reported maternal parenting behavior, Wilks’s λ = .897, *F*(6, 216) = 2.01, *p* = .07. Post hoc analysis showed that adolescents with NSSI reported significantly less maternal warmth and support than NC adolescents (*p* < .01, *d* = 0.64). No significant difference was found for maternal warmth between the NSSI and CC group (*p* > .05) or between the CC and NC group (*p* > .05). The adolescents did not differ in their reports regarding maternal psychological control or maternal behavioral control (demands, rules, and discipline). A significant difference emerged in adolescent-reported paternal parenting behavior, Wilks’s λ = .874, *F*(6, 194) = 2.26, *p* < .05. NC adolescents reported the most paternal warmth and support, followed by NSSI and CC adolescents. Post hoc comparisons between the NSSI and NC group (*p* = .07) and between the CC and NC group (*p* = .06) were nonsignificant. CC adolescents reported the most paternal psychological control, followed by the NSSI and NC group. But the post hoc analysis showed no significant differences between the NSSI and NC group (*p* = .11) or between the CC and NC group (*p* = .07). The adolescents did not differ in their reports regarding paternal behavioral control.

**Table 1:** Means (and standard deviations) of the Zurich Short Questionnaire on Parental Behavior and effect sizes (Cohen’s d) for group comparisons

Group	Warmth/support *M* (*SD*)		Psychological control *M* (*SD*)		Behavioral control *M* (*SD*)	
	Maternal	Paternal	Maternal	Paternal	Maternal	Paternal
Adolescents						
NSSI (*n* = 45)	28.30 (10.26)	29.27 (9.16)	11.95 (6.73)	10.61 (5.30)	15.71 (3.91)	14.73 (4.71)
CC (*n* = 27)	32.83 (7.43)	28.47 (9.03)	11.08 (5.22)	11.33 (6.41)	15.11 (4.26)	12.83 (4.71)
NC (*n* = 44)	34.10 (7.80)	33.48 (5.98)	9.42 (6.28)	7.99 (4.87)	15.54 (3.62)	13.11 (4.44)
Mothers						
NSSI (*n* = 36)	33.93 (6.89)		8.86 (4.88)		16.31 (5.98)	
CC (*n* = 22)	34.44 (4.27)		5.59 (2.81)		15.05 (3.62)	
NC (*n* = 34)	33.87 (5.01)		6.47 (4.24)		14.94 (3.41)	
Fathers						
NSSI (*n* = 9)		33.48 (3.15)		8.25 (4.77)		12.75 (3.73)
CC (*n* = 5)		30.40 (6.80)		6.40 (6.19)		12.80 (3.70)
NC (*n* = 10)		33.66 (5.87)		8.50 (3.75)		13.00 (2.00)
Cohen’s *d (adolescent self-report)*						
NSSI vs. CC	0.49	0.09	0.14	0.13	0.15	0.41
NSSI vs. NC	0.64	0.55	0.39	0.52	0.05	0.36
CC vs. NC	0.17	0.70	0.29	0.62	0.11	0.06

*NSSI* Adolescents with nonsuicidal self-injury; *CC* clinical controls (adolescents with other mental disorders); *NC* nonclinical controls (adolescents without mental disorders)

### Parent–adolescent agreement

The results of the mother–adolescent and father–adolescent agreement over all groups are reported in Table 2. All three groups showed low mother–adolescent agreement regarding maternal warmth and support (*r* = .24 to .31). In the NSSI and CC group, mothers rated the warmth and support they give their children as higher than the adolescents rated them themselves (NSSI group Cohen’s *d* = 0.64, CC group *d* = 0.26). No significant differences in the MANOVA were revealed in mothers’ reports of their own parenting behavior, Wilks’s λ = .891, *F*(6, 174) = 1.72, *p* = .12. Mothers’ reports on psychological control were lower than adolescents’ reports (NSSI group Cohen’s *d* = 0.52, CC group *d* = 1.30, NC group *d* = 0.54). The mother–adolescent agreement on maternal psychological control was low in the NSSI group (*r* = .25) and better in the CC (*r* = .58) and NC (*r* = .52) group, but these differences were not significant. Mothers did not differ in their reports on behavioral control (*p* > .05). The mother–adolescent agreement on maternal behavioral control was highest in the CC group (*r* = .46), followed by the NC (*r* = .29) and the NSSI (*r* = .19) group.

**Table 2:** Mother–adolescent and father–adolescent agreement on dimensions of parenting behavior (Pearson’s correlation) over all groups

Dimension	NSSI	CC	NC	*z* scores		
				NSSI vs. CC	NSSI vs. NC	CC vs. NC
Mother–child agreement	*n* = 36	*n* = 22	*n* = 34			
Maternal warmth/support	0.24	0.25	0.31	−0.04	−0.30	−0.22
Maternal psychological control	0.25	0.58**	0.52**	−1.41	−1.28	0.30
Maternal behavioral control	0.19	0.46*	0.29	−1.06	−0.43	0.68
Father–child agreement	*n* = 9	*n* = 5	*n* = 10			
Paternal warmth/support	0.48	0.70	0.39	−0.42	0.20	0.57
Paternal psychological control	0.28	0.34	0.39	−0.08	−0.22	−0.07
Paternal behavioral control	0.16	0.44	0.84**	−0.38	−1.91	−0.93

*NSSI* Adolescents with nonsuicidal self-injury; *CC* clinical controls (adolescents with other mental disorders); *NC* nonclinical controls (adolescents without mental disorders)

*p < .05, **p < .01

Father–adolescent agreement regarding paternal warmth and support ranged from *r* = .39 to .70. Similar to the mothers, fathers in the NSSI and CC group rated the warmth and support in their own parenting behavior as higher than adolescents rated them themselves (NSSI group Cohen’s *d* = 0.50, CC group *d* = 0.23). The father–adolescent agreement on paternal psychological control was quite low in all groups (*r* = .28 to .39). A high level of father–adolescent agreement was found for paternal behavioral control in the NC group. Fathers of the three groups did not differ in their reports on their own parenting behavior, Wilks’s λ = .839, *F*(6, 36) = .55, *p* = .77.

### Family situation

The majority (88.9 %) of adolescents with NSSI lived together with both parents before the inpatient stay. One adolescent lived in sheltered accommodation, another one had been previously treated in a child and adolescent psychiatry unit, and a third one lived in a foster family. In the CC group, 74.1 % of the parents were married, thus more than in the NSSI group (64.4 %) and the NC group (52.3 %). Eight adolescents in the NSSI group, four adolescents in the CC group, and two adolescents in the NC group reported parental mental illness.

### Maternal psychopathology and parental satisfaction

The maternal DASS-21 scores were all in the normal range (see Table 3). However, the three groups differed significantly regarding maternal psychopathology, Wilks’s λ = .814, *F*(6, 150) = 2.72, *p* < .05. Post hoc analysis showed that mothers in the NSSI group reported significantly more depressive symptoms (*p* < .05, *d* = 0.7), anxiety symptoms (*p* < .05, *d* = 0.7), and stress symptoms (*p* < .01, *d* = 0.86) than mothers in the NC group. These differences did not emerge between mothers of the NSSI and CC group (*p* > .05). In the NSSI group, 50 % of the mothers felt that they had a lot of nervous energy and found it hard to “wind down” (33.3 %) and relax (25 %).

**Table 3:** Parents’ mean scores (and standard deviations) on the DASS-21 and effect sizes (Cohen’s d) for group comparisons

Group	Overall score		Depression	Anxiety	Stress
	*M* (*SD*)	Cohen’s *d*	*M* (*SD*)	*M* (*SD*)	*M* (*SD*)
Mothers					
NSSI (*n* = 36)	26.26 (19.60)		7.38 (8.80)	7.16 (10.8)	13.06 (7.18)
CC (*n* = 22)	17.80 (16.06)		5.00 (5.78)	2.62 (5.22)	11.09 (7.22)
NC (*n* = 34)	11.71 (11.73)		2.59 (4.02)	2.08 (2.78)	7.52 (5.85)
NSSI vs. CC		0.47			
NSSI vs. NC		0.91			
CC vs. NC		0.46			
Fathers					
NSSI (*n* = 9)	28.44 (16.66)		6.66 (5.92)	5.33 (6.40)	16.44 (5.90)
CC (*n* = 5)	14.00 (15.17)		3.20 (3.63)	1.60 (2.19)	9.20 (9.86)
NC (*n* = 10)	11.11 (14.04)		2.60 (5.74)	1.40 (2.12)	6.80 (6.61)
NSSI vs. CC		0.96			
NSSI vs. NC		1.20			
CC vs. NC		0.22			

*DASS-21* Depression Anxiety Stress Scale-21; *NSSI* Adolescents with nonsuicidal self-injury; *CC* clinical controls (adolescents with other mental disorders); *NC* nonclinical controls (adolescents without mental disorders)

A significant difference emerged in the overall score of the PSS between mothers of the three groups, Wilks’s λ = .648, *F*(10, 170) = 4.12, *p* < .01. Post hoc analyses indicated that mothers in the NSSI group reported less parental satisfaction than mothers in the CC group (*p* < .05, *d* = 0.61) and mothers in the NC group (*p* < .01, *d* = 0.8). As reported in Table 4, mothers of adolescents with NSSI scored highest on the four subscales of the PSS compared to mothers of the control groups (CC and NC). Their adolescent’s behavior was rated as predominantly embarrassing and stressful by 36.1 % of mothers in the NSSI group, 13.6 % of mothers in the CC group, and 8.8 % in the NC group. The percentage of mothers who worried if they were doing enough for their children was 69.4 % in the NSSI group, 45.5 % in the CC group, and 35.3 % in the NC group.

**Table 4:** Parents’ mean scores (and standard deviations) on the PSS and effect sizes (Cohen’s d) for group comparisons

Group	Overall score		Parental rewards	Parental stressors	Lack of control	Parental satisfaction
	M (*SD*)	Cohen’s *d*	M(*SD*)	M(*SD*)	M(*SD*)	M(*SD*)
Mothers						
NSSI (*n* = 36)	41.95 (9.13)		10.33 (3.06)	17.92 (6.37)	4.94 (1.84)	6.67 (1.80)
CC (*n* = 22)	36.73 (7.98)		9.36 (3.06)	16.18 (4.24)	4.32 (1.49)	5.41 (1.40)
NC (*n* = 34)	34.93 (8.54)		8.88 (2.97)	15.37 (4.59)	4.44 (1.78)	4.38 (1.84)
NSSI vs. CC		0.61				
NSSI vs. NC		0.80				
CC vs. NC		0.22				
Fathers						
NSSI (*n* = 9)	42.50 (8.73)		11.00 (1.69)	17.50 (5.04)	5.75 (2.43)	6.00 (1.60)
CC (*n* = 5)	37.60 (5.98)		8.80 (5.17)	16.40 (3.78)	4.80 (0.84)	5.80 (0.84)
NC (*n* = 10)	35.90 (6.71)		10.70 (2.31)	14.70 (3.02)	4.60 (1.65)	4.40 (1.43)
NSSI vs. CC		0.67				
NSSI vs. NC		0.90				
CC vs. NC		0.28				

*PSS* Parental Stress Scale; *NSSI* Adolescents with nonsuicidal self-injury; *CC* clinical controls (adolescents with other mental disorders); *NC* nonclinical controls (adolescents without mental disorders)

### Paternal psychopathology and parental satisfaction

As reported in Table 3, fathers of adolescents with NSSI showed mild stress symptoms in the DASS-21. The three groups did not differ regarding paternal psychopathology, Wilks’s λ = .674, *F*(6, 36) = 1.31, *p* = .28. However, post hoc analyses indicated that parents in the NSSI group reported more stress symptoms than parents in the NC group (*p* < .05, *d* = 0.9). The paternal depression and anxiety scores in the NSSI group were in the normal range. The paternal DASS-21 scores in the control groups (CC and NC) were all in the normal range. In the NSSI group, most fathers felt that they had a lot of nervous energy (88.9 %) and they found it hard to “wind down” (44.4 %) and relax (44.4 %).

Table 4 also presents the paternal scores of the PSS. No significant group difference was found for father-reports on the PSS, Wilks’s λ = .469, *F*(10, 32) = 1.47, *p* = .20). Nevertheless, fathers of adolescents with NSSI showed the highest stress scores. It should be noted that the sample size of participating fathers was very small.

## Discussion

The aim of the present study was to examine the parenting behavior in families of adolescents with NSSI disorder, adolescents with other mental disorders, and adolescents without mental disorders. Results indicated only a marginally significant group difference in adolescent-reported maternal parenting behavior. Post hoc tests showed that this was due to lower levels of maternal warmth and support reported by adolescents with NSSI compared to NC adolescents. This is in line with previous research showing that adolescents with NSSI compared to NC adolescents experience the relationship with their parents as being characterized by failed protection, high levels of negative affect, and low levels of positive affect and cohesiveness [12, 22]. However, given the omnibus test was only marginally significant, this result should be interpreted with caution. The NSSI and NC group differed in adolescent-reported maternal warmth and support but not in adolescent-reported paternal warmth and support. Nevertheless, adolescents in the NC group reported more paternal warmth and support than adolescents in the NSSI group. The sample size of participating fathers was small (24 fathers, vs. 92 mothers); therefore, the power was limited. Both mothers and fathers rated the warmth and support they give to their children as higher than the adolescents rated them themselves. Adolescents in the present study showed a low level of parent–adolescent agreement on parenting behaviors. This is in line with previous studies indicating poor agreement between parents and their children when reporting on parenting behavior and family relationships [37, 38].

In contrast to Baetens et al.’s [23] findings, our results did not show a significant group difference in adolescent-reported parental psychological control or parental behavioral control. The inconsistent results regarding parental behavioral control might be explained by the different measures used to assess behavioral control and hence the different definitions of behavioral control. In the Parental Behavior Scale used by Baetens et al. [23], behavioral control is defined as harsh punishment and neglect, whereas behavioral control in the ZKE, which we used, refers to demands, rules, and discipline. Similar to Baetens et al. [23] we found no significant differences in parent-reports of parental behaviors. A further difference between the Baetens et al. [23] study and the present study is that mothers of adolescents with NSSI in this study differed significantly from mothers of the NC group in their reports on parental stress. This may be due to the differences in the examined samples. Our sample consisted of inpatient adolescents with repetitive NSSI, whereas Baetens et al. [23] investigated a nonclinical sample of adolescents. Similar to the results of Morgan et al. [24], parents of adolescents with NSSI in the present study reported more parental stress and less parental satisfaction than parents of both control groups (CC and NC). In addition, there was a significant difference in the number of diagnoses between adolescents with NSSI and CC adolescents. Parents of adolescents with NSSI may be more stressed about their child than parents of CC adolescents because of the number of comorbid disorders. The percentage of mothers who worried if they were doing enough for their children was highest in the NSSI group. Furthermore, mothers of adolescents with NSSI reported more depressive, anxiety, and stress symptoms than mothers in the NC group, and fathers of adolescents with NSSI showed elevated stress symptoms in the DASS-21. The psychopathology of parents of adolescents with NSSI has to be further investigated. Especially, since genetic predisposition for high emotional reactivity and familial hostility and criticism are distal risk factors for NSSI, as proposed by Nock’s [39] integrated theoretical model of the development and maintenance of NSSI. Our results indicate that the development and maintenance of NSSI may not only be influenced by familial hostility and criticism but also by a lack of warmth and support. As distal risk factors also influence interpersonal vulnerability factors, future studies should address the question, if poor verbal and social skills influence the parent-adolescent agreement on parenting behavior.

The results of the present study should be interpreted in the context of some limitations. The current study cannot explain the direction of effects between NSSI and parenting behaviors; this should be investigated in future prospective longitudinal studies. Only with prospective longitudinal designs it is possible to detect causalities in these very different complex parent–child interactions. Given that post hoc analyses were interpreted following a marginally significant omnibus tests, replication is needed. The sample consisted of female adolescents admitted to an inpatient child and adolescent psychiatric unit and thus may not generalize to other samples. Male adolescents with NSSI should be included in further studies. It is uncertain if the reported group differences in the mother–daughter relationship would emerge in male adolescents, as well. Bureau et al. [22] did not find any association between parent–child relationship dimensions and NSSI in male adolescents. In addition, factors that influence parent–child agreement (e.g., negative cognitive bias) as well as response biases (e.g., social desirability) should be included in further studies.

Strengths of the study were the use of the *DSM-5* diagnostic research criteria for NSSI and the use of a multi-informant approach, assessing adolescents and their parents, and the inclusion of a clinical control group of adolescents with mental disorders without NSSI.

Considering the high proportion of adolescents (80 %) who report conflicts within the family as triggers for NSSI, therapy programs for adolescents with NSSI should focus on improving family communication and interaction. Parents and therapists should be aware of parenting difficulties that are associated with NSSI. Information and skills needed for adequate parenting can be addressed in parent programs to reduce parental stress. So far, only a few treatment studies of dialectical behavior therapy [40, 41] and mentalization-based treatment [42] for adolescents with self-injurious behavior or borderline symptoms have included parents in therapy. A tendency toward amelioration was found for family and peer contacts [40]. The inclusion of parents in interventions for adolescents with NSSI (e.g., dialectical behavior therapy) might improve family functioning. Adding aspects from the work group of Fruzzetti [43, 44], the explicit training of emotion-validating communication and social problem solving might improve outcome for patients and strengthen family cohesion. Given the high psychosocial burden and the variety of professionals involved in treatment, aspects of multisystemic therapy (MST) might also be helpful. Huey et al. [45] showed that MST can reduce suicide attempts and improve family relationships. Considering the long-term course of NSSI and its high risk of suicide attempts and suicide and the extremely good and long-lasting effects of MST [46], it might be very useful for improving concrete family interaction. It might be helpful to combine skills training and cognitive behavioral therapy interventions (e.g., mindfulness, communication, problem solving, stress tolerance, emotion regulation) with classic family therapeutic interventions [45, 47, 48]. It will be important to develop guidelines for deciding between different treatments with multiple variations and levels of family-centered interventions. Taking into account the high burden on the family there is an imminent need for the development and implementation of evidence-based family therapeutic interventions to improve and save the mental health of all family members.
